# Olfactory, Taste, and Photo Sensory Receptors in Non-sensory Organs: It Just Makes Sense

**DOI:** 10.3389/fphys.2018.01673

**Published:** 2018-11-27

**Authors:** Nicholas M. Dalesio, Sebastian F. Barreto Ortiz, Jennifer L. Pluznick, Dan E. Berkowitz

**Affiliations:** ^1^Department of Anesthesiology and Critical Care Medicine, Johns Hopkins University, Baltimore, MD, United States; ^2^Department of Otolaryngology/Head & Neck Surgery, Johns Hopkins University, Baltimore, MD, United States; ^3^Department of Physiology, Johns Hopkins University, Baltimore, MD, United States

**Keywords:** G-protein couple receptor, bitter taste receptor, opsins, olfactory receptor (OR), sensory receptor

## Abstract

Sensory receptors that detect and respond to light, taste, and smell primarily belong to the G-protein-coupled receptor (GPCR) superfamily. In addition to their established roles in the nose, tongue, and eyes, these sensory GPCRs have been found in many ‘non-sensory' organs where they respond to different physicochemical stimuli, initiating signaling cascades in these extrasensory systems. For example, taste receptors in the airway, and photoreceptors in vascular smooth muscle cells, both cause smooth muscle relaxation when activated. In addition, olfactory receptors are present within the vascular system, where they play roles in angiogenesis as well as in modulating vascular tone. By better understanding the physiological and pathophysiological roles of sensory receptors in non-sensory organs, novel therapeutic agents can be developed targeting these receptors, ultimately leading to treatments for pathological conditions and potential cures for various disease states.

## Introduction

The ability of an organism to sense and respond to its environment is critical to its survival. Indeed, the capacity to see, hear, smell, taste, or feel has been intrinsically tied to the evolution of all organisms, from the more nuanced responses of single cell migration along chemical gradients, to translation of light signals into visual images of the world around us. These functions are performed by receptors that are activated by diverse chemicals, a wide variety of mechanical stimuli, and light. Most of these receptors belong to a family of G-protein-coupled receptors (GPCRs) that transduce signals from outside of the cell/organism to stimulate signaling processes within the cell. These signaling pathways lead to responses such as changes in electrical activity, motion, and other physiologic functions. The responses are initiated when specific agonists bind to the receptor, causing a conformational change in the seven-transmembrane structure that is followed by G-protein coupling or decoupling. Ultimately this leads to activation of enzymes or channels resulting in secondary messenger production. To prevent runaway signaling, mechanisms have evolved to halt or dampen the process. These mechanisms include G-protein receptor kinases (GRKs) that phosphorylate the carboxyl tail of the receptor, bind arrestins, and promote uncoupling of the receptor, thereby putting a break on its function. Thus, a sophisticated process has evolved that allows an organism to alter its function in response to external stimuli.

Our classic understanding of sensory receptors has been that they are confined to the sensory organs in which they were initially identified: olfactory receptors in the nose, taste receptors on the tongue, and light receptors in the retina. However, this idea was quickly altered after olfactory receptors were identified in extra-olfactory tissue and cells, specifically sperm (Parmentier et al., 1992). Indeed, it has been suggested that the egg releases chemical chemoattractants to which the sperm are attracted (the sperm literally “smell out” the egg) (Spehr et al., 2003). A growing body of literature describes unexpected functions for sensory receptors in other organs, including regulation of vascular relaxation in blood vessels by opsins and involvement of taste receptors in the airway. In this review we present current knowledge about the function of sensory receptors found outside their classical sensory organs.

## Taste receptors

Taste sensation can be divided into five distinct subtypes, including sweet, bitter, salty, sour, and umami, which is described as savory and the taste for glutamate. Ion channels transduce both sour and salty taste sensations, whereas, bitter, sweet, and umami sensations utilize GPCRs. Two different types of taste GPCRs have been identified: TAS1R encodes for sweet and umami receptor proteins, and TAS2R encodes for bitter receptor proteins. TAS1R receptors were found in mice that have a specific locus (*Sac*) and aversions to the bitter compound saccharin (Fuller, 1974). TAS1R receptors have three subunits, TAS1R1, TAS1R2, and TAS1R3, which oligomerize to make sweet (TAS1R1 + TAS1R2) and umami (TAS1R1 + TAS1R3) receptors. TAS2R genes were discovered by two groups after the release of human genome sequences in the year 2000 (Adler et al., 2000; Matsunami et al., 2000). Adler et al. discovered TAS2R1 when examining human chromosome 5 linked to perception of the bitter compound 6-n-propyl-2-thiouracil (PROP) (Adler et al., 2000). Matsunami et al. discovered the sucrose octaacetate aversion (*Soa*) locus on human chromosome 12 and *TAS2R* genes that were similar to a region of mouse chromosome 6. TAS2Rs have as many as 36 subunits depending on the animal.(Matsunami et al., 2000) Sour perception has a number of candidate receptors including HCN1 and HCN4; however, it is primarily sensed via acid-sensing ion channel 2 (ASIC2) (Holzer, 2009; Ishimaru and Matsunami, 2009). Recent publications have shown that taste receptors are expressed and functional in many different organ systems and not restricted to the oral cavity as previously believed, as summarized in Table 1. In addition, their chemosensory properties provide many more functions than taste perception.

**Table 1:** Selected taste, olfactory, and photoreceptors with suggested functions outside of their natural sensory organ in different animal species.

**Name**	**Alternative names**	**Respiratory system**	**GI system**	**GU system**	**CV system**	**CNS**	**Immune system**	**Skin**	**Adipose tissue**	**Retina (non-visual)**	**Others**	**References**
**TASTE RECEPTORS**												
TAS1R2/TAS1R3	Sweet receptors	H, M	H, M	M		M	H, M	H	H, M			McLaughlin et al., 1992; Hofer et al., 1996; Höfer and Drenckhahn, 1999; Jang et al., 2007; Margolskee et al., 2007; Kidd et al., 2008; Mace et al., 2009; Ren, 2009; Taya et al., 2009; Hass et al., 2010; Elliott et al., 2011; Kojima and Nakagawa, 2011; Simon et al., 2013; Kojima et al., 2014; Lee and Cohen, 2014a; Shirazi-Beechey et al., 2014; Malki et al., 2015; Song et al., 2016
TAS1R1/TAS1R3	Umami receptors		H, M		H, M	M	H, M				testis	Taniguchi, 2004; Meyer et al., 2012; Foster et al., 2013; Kendig et al., 2014; Lee et al., 2014; van Avesaat et al., 2015
TAS2R10/TAS2R14		H, M	H		H		H					Rozengurt et al., 2006; Deshpande et al., 2010; Orsmark-Pietras et al., 2013
TAS2R38		H, M		H, M			H,M	H			placenta	Shah et al., 2009; Lee et al., 2014; Malki et al., 2015; Maurer et al., 2015; Marcinek et al., 2016; Ortiz et al., 2016
TAS2R50					H, M							Foster et al., 2013
TAS2R105				H, M							testes	Liu et al., 2015; Zhai et al., 2016
TAS2R108		M	M		M	M					testis	Singh et al., 2011; Krasteva et al., 2012; Foster et al., 2013; Xu et al., 2013; Kok et al., 2018
TAS2R131			M				M					Voigt et al., 2012; Prandi et al., 2018
**PHOTORECEPTORS**												
Opsin 1-S	Short-wavelength							H, Re				Tsutsumi et al., 2009; Fulgione et al., 2014; Haltaufderhyde et al., 2015
Opsin 1-LM	Long/medium wavelength							H				Tsutsumi et al., 2009
Opsin 2	Rhodopsin			H, M				H, C			F (larvae)	Tsutsumi et al., 2009; Shen et al., 2011; Bellono et al., 2013, 2014; Haltaufderhyde et al., 2015; Pérez-Cerezales et al., 2015; Buscone et al., 2017
Opsin 3	Encephalopsin, panopsin	H		H, M	H, M, R	M	H	H		H		Blackshaw and Snyder, 1999; White et al., 2008; Haltaufderhyde et al., 2015; Pérez-Cerezales et al., 2015; Buscone et al., 2017; Barreto Ortiz et al., 2018; Regazzetti et al., 2018
Opsin 4	Melanopsin			H, M	H, M, R	H, T		A	H, M	H, P, M, R,T		Provencio et al., 1998; Rollag et al., 2003; Fernandes et al., 2012; Sikka et al., 2014; Pérez-Cerezales et al., 2015; Nissil et al., 2017; Ondrusova et al., 2017; Barreto Ortiz et al., 2018
Opsin 5	Neuropsin			H, M		H		H		H, M		Kojima et al., 2011; Buhr et al., 2015; Haltaufderhyde et al., 2015; Pérez-Cerezales et al., 2015
RHH	Peropsin							H				Toh et al., 2016
**OLFACTORY RECEPTORS**												
OR51E2	PSGR, mouse: Olfr78, MOR18- 2, MOL2.3, rat: Olr59	H, M	M, R	H, M				H		H	H (retinal pigment epithelial cells)	Neuhaus et al., 2009; Spehr et al., 2011; Matsueda et al., 2012; Pluznick et al., 2013; Rodriguez et al., 2014; Sanz et al., 2014; Cao et al., 2015; Chang et al., 2015; Fleischer et al., 2015; Wiese et al., 2015; Aisenberg et al., 2016; Gelis et al., 2016, 2017; Matsubara et al., 2017
OR1D2		H										Kalbe et al., 2016a
OR2AG1		H										Kalbe et al., 2016a
OR2J3		H	H									Kalbe et al., 2016b, 2017
OR51B4			H									Weber et al., 2017
OR1A1			H									Wu et al., 2015
Olfr544			M									Kang et al., 2015; Wu et al., 2017
OR17-4				H								Spehr et al., 2003, 2004; Neuhaus et al., 2006
Olfr1393				M								Shepard et al., 2016
OR51E1				H	H							Maßberg et al., 2016; Jovancevic et al., 2017a
OR10J					H							Kim et al., 2015
OR2AT4								H				Busse et al., 2014
MOR23	Olfr16										M (muscle)	Griffin et al., 2009; Pichavant et al., 2016

*A, amphibian; CNS, central nervous system; C, cephalopod; CV, cardiovascular; F, fly; GI, gastrointestinal; GU, genitourinary; H, human; M, mouse; P, primate; R, rat; Re, reptile; T, Teleost Fish*.

### Intracellular receptor signal transduction mechanisms

Both TAS1R and TAS2R utilize similar GPCR intracellular signaling effectors, including the heterotrimeric G-protein α-gustducin; phospholipase C (PLC)-β2, which produces inositol 1,4,5-triphosphate (ITP); and diacylglycerol (DAG). Calcium (Ca^2+^) activation of the transient receptor protein (TRP) TRPM5 cation channel (Pérez et al., 2002; Ruiz, 2003) causes Na^+^ influx, leading to depolarization of the cell. All of these signal cascade components are commonplace for chemosensory transduction. Unlike TAS2R receptors, TAS1Rs cause intracellular signaling via gap junctions and paracrine communications that cause adjacent cells to translocate proteins for glucose transportation (Welcome et al., 2015). In fact, it was the identification of α-gustducin (Hofer et al., 1996), and later taste receptors themselves, in brush cells located in the stomach and intestine that confirmed the presence of taste receptors outside the mouth. Zancanaro et al. (1999) was first to discover gustducin-secreting cells in nasal and upper airway tissues, where they are instrumental in airway immunity. The mechanisms for downstream signaling of TAS2Rs are still not well understood. Questions still unanswered include (1) how TRPM5 is activated by Ca^2+^, (2) downstream intracellular effects of Ca^2+^ influx, and 3) how taste receptor cells depolarize.

### Bitter taste receptors outside classic sensory organs

Bitter taste receptors (TAS2Rs) are divided into as many as 36 subunits depending on the animal (over 25 subunits in humans), many of which have been discovered in areas outside the gustatory system, including the respiratory, gastrointestinal, genitourinary, cardiovascular, thyroid, musculoskeletal, immune, and central nervous systems.

#### Airway

**Figure 1:**
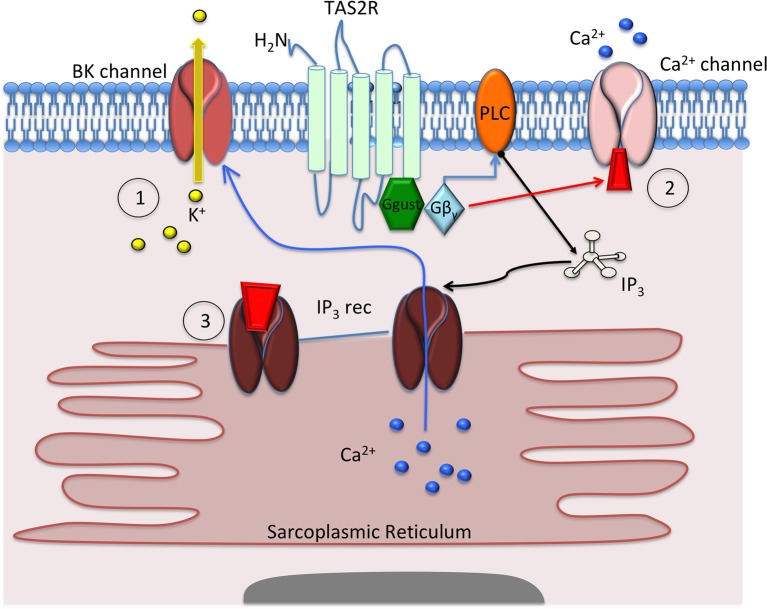
Mechanisms proposed to explain how bitter taste receptor (TAS2R) agonists cause airway smooth muscle (ASM) cell relaxation. Deshpande et al. (1) proposed that activation of the TAS2R caused activation of gusticin, followed by Gβγ and phospholipase C (PLC) subunits, to activate inositol triphosphate (IP3), which in turn activates the IP3 receptor to release calcium (Ca^2+^) from the sarcoplasmic reticulum. This release of calcium causes opening of the BK_Ca_-channel and efflux of potassium (K^+^) that hyperpolarizes and relaxes the ASM cell. Zhang et al. (2) proposed that TAS2R activation leads to small, incremental increases in Ca^2+^, which causes closure of membrane Ca^2+^ channels. This closure inhibits Ca^2+^ influx, cell depolarization, and cell contraction, leading to cellular relaxation. Tan et al. (3) proposed that TAS2R activation causes a change in Ca^2+^ sensitivity and/or changes to Ca^2+^ oscillation that inhibit Ca^2+^ release from the sarcoplasmic reticulum, thereby inhibiting cellular contraction.

Bitter taste receptors in the respiratory system have been the most studied. They can be found on ciliated epithelial, solitary chemosensory, ciliated brush, and airway smooth muscle (ASM) cells. When activated, TAS2Rs cause an increase in ciliary beat frequency (Shah et al., 2009) and mucociliary clearance (Ortiz et al., 2016) as well as release of nitric oxide from ciliated cells and release of antimicrobial proteins (Lee and Cohen, 2016) from solitary chemosensory cells. Within ASM cells, activation of TAS2R-10,−14, and−31 has been shown to cause relaxation and bronchodilation (Deshpande et al., 2010; An et al., 2012). Interestingly, bitter taste receptors mediate relaxation but cause a seemingly paradoxical increase in intracellular calcium upon activation. Although increased calcium typically leads to contraction, bitter taste receptor-mediated increases in calcium cause these cells to relax. This calcium signaling is a complex process, with potential mechanisms ranging from hyperpolarization of the cell via calcium-mediated potassium channels (Deshpande et al., 2010) to alterations in calcium oscillation (Tan and Sanderson, 2014) or sequestration within the mitochondria (Medler, 2010) (Figure 1). In brush cells, TAS2Rs are instrumental for inducing a breath-hold, mediated by acetylcholine (Ach) as well as in chemosensory cells expressing TAS2Rs that enhance the responsiveness of the trigeminal nerve leading to apnea in mice (Tizzano et al., 2010; Krasteva et al., 2012).

#### Gastrointestinal system

In addition to bitter receptors detecting toxic and potentially poisonous foods, bitter agents within common foods, including coffee, beer, soy, and broccoli, have shown beneficial health effects when regularly consumed by humans. Within the stomach, TAS2R ligands activate expression of the early immediate gene c-fos (an oncogene) in the solitary tract nucleus of the brain via the vagus nerve, causing future food aversion in rats (Hao et al., 2009). Bitter receptors also have been discovered in the stomach and intestine (Reimann et al., 2012; Gu et al., 2015), where expression is modulated by diet. TAS2Rs are present in human gastric epithelial cells and when activated, stimulate gastric acid secretion (Liszt et al., 2017). In the intestine, stimulation of bitter receptors on the enteroendocrine STC-1 cells leads to release of cholecystokinin, which, in turn, stimulates ATP-binding cassette B1 transporter to cause toxins to be expunged from the cellular cytoplasm (Masuho et al., 2005; Jeon et al., 2011). After TAS2R activation, colonic epithelium releases anions that cause fluid to be secreted into the lumen, potentially flushing out bowel irritants (Kaji et al., 2009). In addition, one single nucleotide polymorphism discovered in the TAS2R9 haplotype has been shown to alter glucose metabolism and insulin homeostasis, potentially contributing to the metabolic diseases (Dotson et al., 2008). It has been shown that activation of the TAS2R108 receptor increases fat loss, increases glucose tolerance and insulin sensitivity, as well as normalizes plasma lipids in diet-induced obese mice (Kok et al., 2018). In the mucin-producing goblet cells of the murine colon, the *TAS2R131* gene has been shown to be expressed (Prandi et al., 2018).

#### Genitourinary system

TAS2Rs have been discovered in both the kidney, where TAS2R105 plays a role in the murine kidney, and the detrusor muscle of the bladder, where it causes bladder relaxation when activated in human subjects (Liu et al., 2015; Zhai et al., 2016). The TAS2R agonist chloroquine relaxed carbachol-, potassium chloride-, and electrical field stimulation-induced contractions of human detrusor muscle strips *in vitro*. Denatonium and quinine also decreased carbachol-induced contractions of mouse detrusor muscle strips in a concentration-dependent manner (Zhai et al., 2016).

#### Cardiovascular system

Although TAS2Rs have been discovered in both human and rodent cardiac myocytes, their function is primarily unknown. However, they are believed to “sense” glucose because they are upregulated during starvation (Foster et al., 2014). In addition, TAS2Rs were discovered in both cerebral and mesenteric arteries of mice and humans. Administration of the TAS2R agonists chloroquine and quinine has been shown to relax rat mesenteric arteries (Chen et al., 2017).

#### Central nervous system (CNS)

TAS2Rs have been identified in rodent (Singh et al., 2011; Dehkordi et al., 2012; Voigt et al., 2015) and human brain tissue (Ansoleaga et al., 2013); however, their function is currently unknown.

#### Immune system

In addition to providing innate immune function in the ciliated cells of the nasal epithelium, TAS2R38 has also been discovered on leukocytes, including neutrophils, monocytes, and lymphocytes (Malki et al., 2015; Marcinek et al., 2016). Not only are the receptors located on the cell surface, they have also been detected intracellularly, within lipid vesicles. Neutrophils take up *Pseudomonas aeruginosa*-derived quorum sensing molecule N-(3-oxododecanoyl)-L-homoserine lactone (AHL-12), which binds to the TAS2R38 receptor, creating an AHL-12–TAS2R complex. Antibodies to TAS2R will stimulate the neutrophil, suggesting an instrumental role in phagocytosis of gram-negative bacteria (Maurer et al., 2015).

### Sweet taste receptors

Sweet receptors (TAS1R2 + TAS1R3) signal carbohydrate energy sources and are activated by both natural and artificial (e.g., aspartame, sucralose, and saccharin) sweet substances. In addition to the mouth, sweet receptors are also found in the airway, gastrointestinal track, genitourinary system, CNS, and immune system (Taya et al., 2009).

#### Airway

Sweet receptors are antagonistic to bitter receptors and have a significant inhibitory role in airway mucosal and ciliary function (Lee and Cohen, 2014a). Other sensory systems, including the visual, auditory, and olfactory systems, have sweet receptors that, when activated, will affect taste perception (Höfer and Drenckhahn, 1999; Kidd et al., 2008; Mace et al., 2009).

#### Gastrointestinal system

TAS1R2 and TAS1R3 have been found in both the stomach and intestine (McLaughlin et al., 1992; Hofer et al., 1996; Shirazi-Beechey et al., 2014). In the stomach, the common TAS1R3 subunit of sweet and umami receptors was identified in both brush cells and gastrin-producing enteroendocrine G-cells (Hass et al., 2010). Activation of sweet receptors increases ghrelin release, stimulating the appetite. In the intestine, activation of sweet receptors on enteroendocrine L-cells causes release of glucagon-like peptide and peptide tyrosine tyrosine (also known as peptide YY), which each enhance insulin release from β-cells in the pancreas (Jang et al., 2007; Mace et al., 2007). Sweet receptors facilitate glucose absorption and metabolism by causing translocation of the glucose transporters SGLT1 (sodium–glucose co-transporter 1) and GLUT2 (facilitated glucose transporter 2) into the apical membrane of the intestinal epithelium (Margolskee et al., 2007; Song et al., 2016). In the pancreas, sweet receptors have been discovered in islet and MIN6 cells, a glucose-responsive β-cell. When activated by sucralose, insulin secretion is potentiated, but this potentiation is inhibited when the known sweet receptor antagonist gurmarin is present (Kojima and Nakagawa, 2011; Kojima et al., 2014).

#### Genitourinary system

In the rat urinary bladder, sweet receptors have been found in umbrella cells. Unlike TAS2Rs, TAS1Rs in the bladder lead to bladder contraction when activated (Elliott et al., 2011).

#### CNS

Sweet receptors are present in the hypothalamus (Ren, 2009), where hunger is regulated, as well as in the hippocampus, habenula, cortex, and choroid plexus. TAS1R3 knockout mice express reduced dendritic spine density in the hippocampus and cortex and exhibit alterations in learning, memory, and sociability (Martin et al., 2017). These results suggest that sweet-taste perception plays a role in CNS neuronal functionality.

#### Immune system

All of the TAS1R subunits have been found in different types of leukocytes, including monocytes, natural killer cells, and T and B cells, with the highest concentration of all three subunits located in polymorphonuclear (PMN) cells. Chemotaxis of PMN cells toward saccharin is significantly diminished when TAS1R2 and TAS1R3 receptors are inhibited, specifically by the TAS2R3 subunit antagonist lactisole (Malki et al., 2015). Trehalose, a disaccharide that inhibits TNF-α and IL-1β production, is sensed specifically by the TAS1R3 receptor subunit and was shown to cause a decrease in cytokine production and inflammation (Taya et al., 2009). Both TAS1R2 and TAS1R3 receptors in adipocytes have been shown to be involved in adipose metabolism; however, adipogenesis and attenuation of lipolysis are not dependent solely on these receptors, as alternative “sweet” receptors are believed to play a role (Simon et al., 2013).

### Umami receptors

Umami receptors (TAS1R1 + TAS1R3) are activated by two types of chemicals: amino acids, specifically monosodium glutamate and aspartate, and purine 5′-ribonucleotides, including inosine and guanosine monophosphate (IMP and GMP, respectively). Additional umami receptors include mGluR1 and mGluR4, which are truncated variants of glutamate receptors (Chaudhari et al., 2000). Umami, like sweet taste, is species specific, such that humans perceive L-glutamate as an umami tastant, whereas rodents respond mostly to L-amino acids. Umami receptors have been found in almost all of the same systems as sweet receptors, including the gastrointestinal and immune systems, but are also in the cardiovascular system.

#### Gastrointestinal system

The common subunit for sweet and umami taste receptors, TAS1R3, has been the predominant subunit found in the gastrointestinal system, with several investigators believing that TAS1R3 homo-oligomers exist. When these receptors are activated in enteroendocrine cells in the stomach, they release the appetite-stimulating ligand ghrelin. Receptor activation in the small intestine reduces appetite (van Avesaat et al., 2015) and stimulates colonic peristalsis (Kendig et al., 2014). Umami receptors also have been discovered to activate murine neutrophils, increasing chemotactic migration and reducing the lipopolysaccharide-induced inflammatory response (Lee et al., 2014). TAS1R3 has been discovered in the pancreas (potentially homo-oligomers) (Taniguchi, 2004), and like sweet receptors, is associated with potentiation of insulin secretion (Kojima and Nakagawa, 2011).

#### Cardiovascular system

After a period of starvation, murine myocytes exhibited upregulation of TAS1R1 and TASR3 receptor subunits. Their action was similar to that of TAS2R receptors in the same tissue (Foster et al., 2014).

#### Immune system

Umami taste receptors (TAS1R1/TAS1R3) mRNA is expressed in mouse neutrophils. Tastants, specifically L-alanine and L-serine, increase chemotactic migration of neutrophils as well as reduce the production of TNF-α induced by lipopolysaccharide (Lee et al., 2014).

## Photoreceptors

Opsins are photoreceptive compounds present in a wide variety of animals. They share striking similarities among different species. Their conformation changes from a resting state to a signaling state in response to light, which initiate a signaling cascade that ultimately leads to physiologic changes within the cell. Rhodopsin has been the most studied of all opsins since its discovery in the frog retina in 1877. At that time, it was described as a visual pigment that changed colors from red to translucent when exposed to light (Boll, 1977). It also became the first GPCR to have its crystal structure resolved in 2000 (Palczewski et al., 2000). Extensive research on rhodopsin has revealed that the opsins are actually composed of a protein (the opsin, a seven-transmembrane helical structure) and a light-sensitive chromophore (a vitamin A-based retinaldehyde, most commonly retinal) (Wald, 1936). The specific structure of each opsin determines the wavelength that the chromophore will absorb; therefore small changes near the chromophore binding site result in a variety of opsins that react to different wavelengths (Shichida and Matsuyama, 2009).

### Overview of classic opsin signaling

Early studies on rhodopsin showed that it can bind many isoforms of retinal (9-*cis*, 11-*cis*, 13-*cis*, etc.). Of these, 11-*cis*-retinal is preferentially bound to rhodopsin in the dark, and upon light absorption undergoes isomerization to all-*trans*-retinal. This isomerization causes a structural change that activates the opsin, which in turn activates the G protein. Transducin (G_t_) is the natural heterotrimeric G protein expressed in vertebrate rods and cones, and hundreds of transducin molecules may be activated by each activated opsin, providing a significant signal expansion step. During its activation, the transducin associated GDP is exchanged for GTP, which allows the transducin to dissociate into the GTP-bound α subunit and the βγ complex. The G_α_ subunit propagates the phototransduction signal by interacting with cyclic nucleotide phosphodiesterase (PDE). Upon activation of PDE, the phosphodiester bond of cGMP is broken to produce 5′GMP. The decrease in cGMP concentration causes cyclic nucleotide-gated channels on the cell wall to close, preventing the influx of Ca^2+^ and Na^+^ ions into the cell and causing membrane potential hyperpolarization (Shichida and Matsuyama, 2009).

Since the mechanism of rhodopsin phototransduction was delineated, several other opsins and alternative mechanisms have been discovered that produce a wide variety of physiologic changes specific to the organism, tissue, and cell type. For example, in mammalian intrinsically photosensitive retinal ganglion cells (ipRGCs) and in *Drosophila* photoreceptor cells, the opsins are associated with the G protein G_q_, which interacts with PLCβ and eventually opens TRP channels that enable Ca^2+^ and Na^+^ influx into the cells and membrane depolarization (Xue et al., 2011). The evolution and known mechanisms of opsin signaling have been the focus of other reviews (Terakita, 2005; Shichida and Matsuyama, 2009; Hankins et al., 2014). Here we will focus on the current knowledge of photoreceptors outside of the ocular system and their possible clinical applications. We present in Table 1 a selection of opsins with known expression and/or function outside of the eye.

### Opsin functions outside of the ocular system

Opsins have been demonstrated to have non-image-forming roles in the eye, ranging from circadian rhythm modulation to pupillary light reflex. For example, melanopsin has been identified on the iris sphincter muscle, where light directly increases sphincter muscle tension even in the presence of cholinergic antagonists (Wang et al., 2017a). Other non-image-forming functions of opsins in the ocular system and some of their roles outside of the eye have been summarized by Leung and Montell (2017).

#### Skin

Several studies have shown that opsins are present in the skin of a wide variety of animals. In fact, melanopsin was first discovered in frog dermal melanophores and was shown to be more closely related to invertebrate opsins (especially octopus rhodopsin) than to the rhodopsin found in the frog's own eyes (Provencio et al., 1998). Skin from cephalopods such as cuttlefish, octopus, and squid have been shown to express opsins and other phototransduction components, particularly rhodopsin and G_qα_, at the mRNA and protein levels (Mäthger et al., 2010; Kingston et al., 2015), though their functionality remains to be tested. Gecko camouflaging by short-term skin darkening was found to be due specifically to light sensitivity in the belly and flank skin, where expression of short-wavelength-sensitive opsin 1 was confirmed (Fulgione et al., 2014).

Peropsin (RHH) expression has been confirmed in human skin, specifically in human epidermal keratinocytes, where it contributes to an all-*trans* retinal-dependent violet-light-induced calcium flux (Toh et al., 2016). RT-PCR also showed short-wavelength-sensitive opsin, rhodopsin, encephalopsin/panopsin, and neuropsin (OPN1-S, OPN2, OPN3, OPN5, respectively) to be expressed in keratinocytes and in human epidermal melanocytes (HEMs) (Haltaufderhyde et al., 2015). However, only OPN2 and OPN3 were found to encode full-length proteins and their functionality has not been tested (Haltaufderhyde et al., 2015). Protein expression of OPN2, OPN1-S, and OPN1-LM (long/medium wavelength-sensitive cone opsin) has been confirmed in human epidermis via immunohistochemistry, with the OPN1-LM being localized in the basal layer deeper in the tissue (Tsutsumi et al., 2009).

Interestingly, rhodopsin was found to contribute to ultraviolet-A (UVA)-induced Ca^2+^ mobilization in HEMs, suggesting that it is part of the phototransduction pathway of UVA-induced melanin synthesis. This finding suggests rhodopsin, which has an absorption maximum of 500 nm when bound to 11-*cis* retinal and 478 nm when bound to 9-*cis* retinal might have an alternate stable state in HEMs that allows it to detect UVA light (320–400 nm) (Wicks et al., 2011). Additional studies demonstrated that exposure of HEMs to physiologic levels of UV radiation activate a retinal-dependent current mediated by Gα_q/11_ and PLCβ. This current was mediated by TRPA1 ion channels, which were found to be activated after Gα_q/11_/PLCβ-induced hydrolysis of PIP_2_ to generate DAG and IP_3_. Intracellular calcium increase therefore resulted both from IP_3_-stimulated intracellular Ca^2+^ release and from PIP_2_-regulated, TRPA1-mediated, extracellular Ca^2+^ influx, resulting in downstream melanin synthesis (Bellono et al., 2013, 2014).

More recently it was shown that blue light (415 nm) induced pigmentation in healthy individuals, an effect not observed with red light (630 nm) (Duteil et al., 2014). Additional studies showed that OPN3 mediates this blue-light-induced melanogenesis in human melanocytes. This process is calcium-dependent and ultimately produces an increase in the melanogenesis enzymes tyrosinase and dopachrome tautomerase, though the phototransduction pathway leading to calcium mobilization remains unexplored (Regazzetti et al., 2018). This discovery could have immediate clinical applications, as it suggests that patients with skin hyperpigmentation could benefit from using sunscreen against broad-spectrum UV supplemented with a filter for short-wavelength blue light (Boukari et al., 2015).

Light therapy is currently being investigated for the treatment of acne. Several studies have shown the potential for LED-delivered light to have antibacterial and anti-inflammatory properties. More recently, 415 nm blue light was found to suppress the proliferation of human primary sebocytes, and 630 nm red light to strongly downregulate lipid production, though the mechanisms of phototransduction remain unknown (Jung et al., 2015).

#### Adipose tissue

The recent discoveries of functional photoreceptors on the skin raise the possibility of other light-induced responses in deeper tissues. Although UV light does not penetrate into deep tissues, a small percentage of visible light does penetrate through the skin to varying depths proportional to its wavelength. Indeed, a recent study on subcutaneous white adipose tissue, which plays an active role in the development of diabetes, obesity, and cardiovascular diseases, revealed that blue light induced an inward current on human and mouse adipocytes that was mediated by melanopsin, PLC, and TRP channels (Ondrusova et al., 2017). Furthermore, daily exposure to blue light induced physiologic changes in the adipocytes, including reduced lipid storage, which could imply a light-induced protective mechanism.

Interestingly, studies have shown that light can affect human adipose-derived stem cells. Blue and green light increased intracellular calcium levels, halted proliferation, and promoted osteoblast differentiation, whereas red and near-infrared (NIR) light stimulated proliferation (Wang et al., 2016). Blue, green, and red light also produced different effects on freshly isolated human adipose tissue-derived stromal vascular fraction cells, which include adipose-derived stem cells. Red light again promoted proliferation, and, interestingly, green and red light promoted vasculogenesis (Wang et al., 2017b).

#### Cardiovascular system

**Figure 2:**
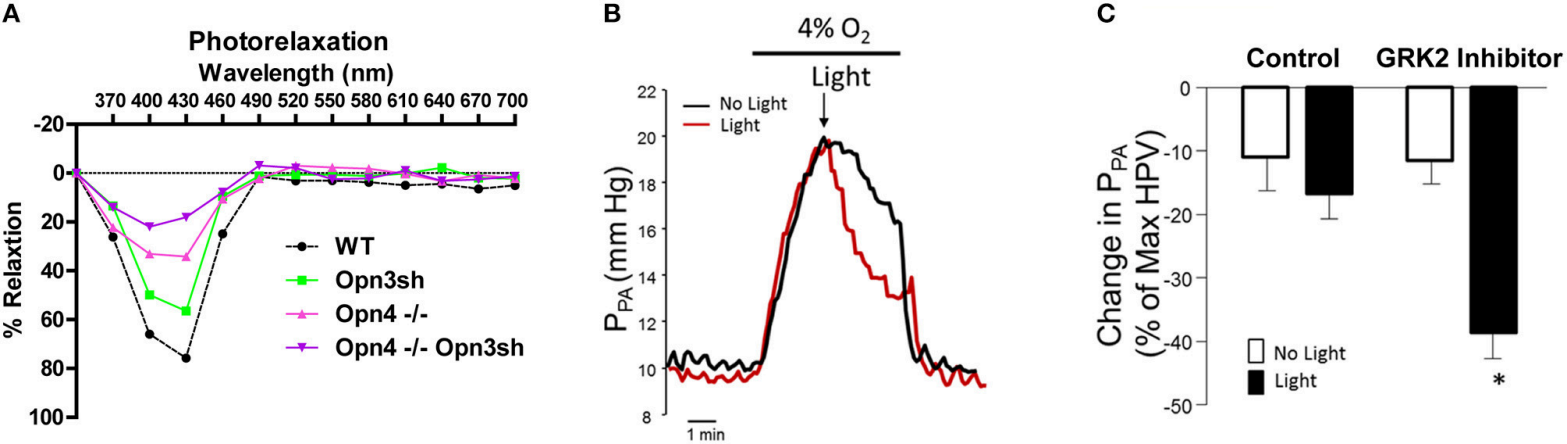
Effects of light exposure on pulmonary artery relaxation. **(A)** Relaxation of control (WT), Opn4 knockout (Opn4^−/−^), and Opn3sh-treated mouse pulmonary arteries exposed to specific light wavelengths in 30 nm increments. **(B)** Representative traces show pulmonary arterial pressure (P_PA_) of isolated perfused rat lungs subject to hypoxia-induced pulmonary hypertension (HPV). Lungs were exposed to blue light with/without GRK2 inhibitor at peak pressure. **(C)** Statistical comparison of changes in P_PA_ after HPV with/without blue light and GRK2 inhibitor. *P < 0.05. Reproduced with permission from Barreto Ortiz et al. (2018).

Although photorelaxation, the light-induced dilation of blood vessels, was first described in 1955 (Furchgott et al., 1955), it was not until 2014 that the effect was shown to be mediated by melanopsin (Sikka et al., 2014). In that study the authors showed that photorelaxation was blue-wavelength-specific and that both pharmacologic inhibition and genetic knockdown of melanopsin (Opn4) ablated the response in the mouse aorta. Interestingly, the response was quickly desensitized, with vessels returning to their constricted state after continued light exposure. Incubation with a G protein coupled receptor kinase 2 (GRK2) inhibitor prevented this desensitization and amplified photorelaxation, suggesting G-protein-mediated phototransduction. More recently, photorelaxation was also shown to involve Opn3 (encephalopsin), as suppression of both Opn3 and Opn4 in mouse pulmonary artery almost completely abrogated blue wavelength (400–460 nm) light-induced vascular relaxation (Figure 2A). The vascular photorelaxation response was shown to be mediated by sGC/cGMP (Barreto Ortiz et al., 2018).

In the presence of GRK2 inhibitor, blue light both reverted and prevented phenylephrine-induced constriction of isolated pulmonary arteries. Furthermore, this treatment ablated single-cell constriction and reversed phenylephrine-induced depolarization of isolated pulmonary arterial smooth muscle cells. More importantly, a phototherapeutic treatment consisting of GRK2 inhibition plus exposure to blue light induced significant vasorelaxation of pulmonary arteries from rats with chronic pulmonary hypertension (PH), even when traditional vasodilatory drugs had only a modest effect. Phototherapy also reduced pulmonary arterial pressure in the lungs of rats subject to acute hypoxia-induced PH (Figures 2B,C), demonstrating the clinical potential of using phototherapy to treat vasoconstrictive diseases such as PH (Barreto Ortiz et al., 2018).

#### CNS

It is now well known that opsins perform numerous non-image-forming functions, with perhaps the most well studied being photoentrainment and circadian rhythm regulation. In mammalian vertebrates, these functions are performed largely by opsins in the eye, as damage to the eyes ablates photoentrainment (Nelson and Zucker, 1981). Photoentrainment is regulated both through light detection in rods and cones and through the effect of melanopsin in intrinsically photosensitive retinal ganglion cells (ipRGCs) (Hannibal and Fahrenkrug, 2002; Panda et al., 2002; Rollag et al., 2003). More recently, neuropsin (OPN5) was shown to be necessary for non-visual retinal photoentrainment in mammals, dictating molecular circadian rhythms *ex vivo* and revealing a role for what was until then an orphan photoreceptor (Buhr et al., 2015). Despite the critical role of the eyes in mammalian photoentrainment, however, the CNS has been shown to play a crucial part in the photoreception that regulates circadian rhythm in a wide range of other animals.

In teleost fish and amphibians, for example, several photoreceptive sites have been identified outside the eyes, including the pineal complex, deep brain, and skin (Peirson et al., 2009). In fact, the pineal complex has been the focus of several photoentrainment studies in non-mammalian vertebrates. In a variety of species, the expression of several opsins has been confirmed, including pinopsin (460–470 nm), vertebrate ancient opsin (460–490 nm), exorhodopsin (498 nm), parapinopsin (370 and 515 nm bi-stable), teleost multiple tissue opsin, and parietopsin (520 nm) (Peirson et al., 2009; Hankins et al., 2014). In zebrafish, the pineal gland contains an endogenous circadian pacemaker and expresses an opsin that is called exorhodopsin because of its similarity to rhodopsin (Mano et al., 1999), though its function has not yet been determined. Other opsins have been found in zebrafish deep brain (Kojima et al., 2000), with melanopsin-mediated phototransduction in neurons shown to mediate zebrafish larvae light-seeking motility (Fernandes et al., 2012). This finding suggests that deep brain opsins also have functions outside of photoentrainment, particularly during development. Indeed, other studies have confirmed the role of opsins during zebrafish embryogenesis and identified vertebrate ancient long opsin in the CNS as a regulator of light-induced motor behavior and neural activity.

*Drosophila* have an alternative entrainment pathway that is mediated by cryptochrome (Cry). Cry is a blue-light-sensitive protein that was found to aid in *Drosophila* photoentrainment and suggested to provide an independent cell-autonomous response to light in ventral lateral brain neurons (Emery et al., 2000). Because silencing of Cry did not completely eliminate circadian photoentrainment, researchers suspected that another photoreceptor also played a role. A recent study revealed a seventh rhodopsin in *Drosophila* (Rh7) that contributes to photoentrainment by circadian pacemaker neurons in the brain, mediating a direct response to violet light (405 nm) (Ni et al., 2017).

These discoveries in a wide variety of animals suggest an adaptation to environmental light in the CNS that might have been lost during mammalian evolution, might still be present as a vestigial system, or might actually remain active in humans and have potential clinical applications. Encephalopsin was the first opsin found outside of the ocular system in the mammalian brain (Blackshaw and Snyder, 1999). It is highly expressed in the paraventricular area of the hypothalamus, specific regions of the cerebral cortex, and even in certain neurons of the spinal cord (Blackshaw and Snyder, 1999; Nissilä et al., 2012). Neuropsin also has been found in the human retina, brain, and testis (Tarttelin Emma et al., 2003). Its activation by light in the UV range stimulates the G-protein G_i_ (Kojima et al., 2011). Melanopsin was also widely expressed at mRNA and protein levels in the human brain (Nissil et al., 2017).

Recent studies have shown that extraocular blue light delivered via the ear canals can influence human brain function and may have antidepressant effects (Jurvelin et al., 2014), though the mechanism of action has not been shown. Furthermore, low-level light therapy, recently renamed photobiomodulation, has been suggested to provide neuroprotection against Parkinson's and other neurodegenerative diseases, and to provide beneficial effects in wound healing after traumatic events and in psychiatric disorders (Schiffer et al., 2009; Hamblin, 2016; Johnstone et al., 2016). The suggested mechanism of action entails increasing cytochrome oxidase and superoxide dismutase activities (Rojas et al., 2008), which increase cytochrome c oxidation, oxygen consumption, and mitochondrial membrane potential, thereby altering electron flow (Rojas and Gonzalez-Lima, 2011). Though these effects are modulated by a photoacceptor and not a photoreceptor *per se*, the wide range of NIR light that has been shown to elicit the beneficial effects of photobiomodulation suggest that other photoacceptors/photoreceptors might be involved (Hamblin, 2016). Moreover, these studies prove that some light can penetrate the skull to produce physiologic responses in the human brain.

#### Unknown and non-light-induced roles of opsins

The OPN3 gene has been implicated in asthma for specific populations. It is expressed in the lung bronchial epithelia and in immune cells, suggesting that it may have a role in asthma susceptibility and immune response modulation (White et al., 2008). How this response is modulated, however, has not been demonstrated. Interestingly, Yim et al. (2018) showed that OPN3 is expressed in bronchial smooth muscle cells and mediates blue-light-dependent bronchial relaxation, a process that is enhanced in the presence of 9-*cis*-retinal.

Perhaps more intriguing is the finding that opsins can serve a non-light-dependent sensing role. It was already known that *Drosophila* larvae move preferentially to regions that have the optimal temperature of 18°C and that this process is mediated by G_q_, PLC, and TRPA1 (Kwon et al., 2008). In 2011 it was discovered that *Drosophila* rhodopsin in fact mediates this response, as genetic silencing of the gene for this protein abolished the response, and introduction of mouse melanopsin restored normal thermotactic behavior (Shen et al., 2011). These findings oppose the dogma that opsin photoreceptors serve only to detect light and opens the possibility for unexplored physiologic functions.

Indeed, a recent study showed that opsins play a role in mouse and human sperm thermotaxis (Pérez-Cerezales et al., 2015). Mammalian sperm is extremely sensitive to temperature changes, and this study showed that opsins modulate temperature-induced movement of both mouse and human spermatozoa. Interestingly, encephalopsin, melanopsin, rhodopsin, and neuropsin were each found to be distinctly localized within specific regions of the spermatozoa. Inhibition of the opsins resulted in decreased sperm thermotaxis in the dark, and thermotaxis was found to be defective in rhodopsin-knockout mice (Pérez-Cerezales et al., 2015).

## Olfactory receptors

Olfactory receptors (ORs) are GPCRs that signal by elevating intracellular cAMP. The OR gene family was first identified by Buck and Axel (Buck and Axel, 1991) and is now known to contain ~1000 members in mice and rats, and ~350 members in humans (Malnic et al., 2004). In the olfactory epithelium, ORs signal using a “labeled line” system—each olfactory sensory neuron expresses one and only one OR; thus an action potential in a particular neuron indicates that the receptor expressed by that neuron has been activated. In addition, ORs in the nose signal combinatorially; that is, rather than each scent being encoded by one neuron, a particular combination of neurons firing at the same time indicates a particular scent. Because of this, the system has the capacity to identify a massive number of odors; although the precise number of odors that humans can discriminate remains unknown, it has been estimated to be as high as 1 trillion (Bushdid et al., 2014; Gerkin and Castro, 2015; Meister, 2015).

When an OR in an olfactory sensory neuron binds to its ligand, it activates an olfactory G-protein (G_olf_, similar to G_s_), which subsequently activates adenylate cyclase 3 (AC3), leading to cAMP production. The resultant influx of cAMP activates cyclic nucleotide-gated channels, ultimately leading to a series of ion fluxes that produce an action potential. It is worth noting that animals which are null for either G_olf_ or AC3 cannot smell (Belluscio et al., 1998; Wong et al., 2000). Because mice at birth are dependent on smell to nurse, these anosmic mice typically die shortly after birth.

Here, we will briefly highlight what is known about the roles of human, murine, or rat receptors in each of the systems listed below, as summarized in Table 1. Because there is a plethora of data on ORs expressed “ectopically,” in this section we will do our best to highlight publications of particular interest. Although we will not be able to cite and discuss every study on ORs outside the nose, an outstanding review focused on the roles of human ORs outside the nose has recently been published (Maßberg and Hatt, 2018). Finally, it is important to note that many studies of ORs have used mice as a model system; however, because this gene family is very large, the identity of human orthologs for murine/rat ORs is often unclear (indeed, it is often unclear whether a human ortholog exists at all). To that end, in the text below we will refer to human ORs except when indicated otherwise; for murine and rat ORs, we will state whether or not a human ortholog is known.

### OR functions outside of the nose

#### Airway

The earliest report of ORs in the airway (Gu et al., 2014) identified them in a unique cell type, pulmonary neuroendocrine cells (PNECs), and suggested that they act as chemosensors in these cells. The study showed that exposure of the lung epithelium to volatile chemicals activates PNECs, leading to changes in the secretion of compounds that affect neighboring cells. Intriguingly, the data also suggested that these processes may be altered in chronic obstructive pulmonary disease. In 2015, Chang et al. (Chang et al., 2015) reported that one OR (Olfr78, which has a clear human ortholog, OR51E2) is expressed in the glomus cells of the carotid body, where it plays a role in the regulation of breathing; however, a recent study failed to confirm a role for Olfr78 in this pathway (Torres-Torrelo et al., 2018). In 2016, two papers described the role of ORs in ASM cells. One of these studies focused on the role of OR1D2 and OR2AG1 and showed that activating these receptors modulated cell contractility (Kalbe et al., 2016a). The other study identified a number of ORs as being expressed in ASM cells, along with G_olf_ and AC3, but focused on the role of OR51E2 (Aisenberg et al., 2016). That study demonstrated that ligands for OR51E2 modulated proliferation and cytoskeletal remodeling and that these effects were dependent on OR51E2 expression. Intriguingly, these effects occurred in ASM from individuals both with and without asthma. Finally, a study in 2017 examined a non-small-cell lung cancer cell line (Kalbe et al., 2017) and found that the OR2J3 agonist helional activates phosphoinositol-3 kinase (PI3K), leading to an increase in intracellular calcium and ERK phosphorylation. Furthermore, helional acts on OR2J3 to induce apoptosis and inhibit cell proliferation, implying that this pathway may have therapeutic benefit.

#### Gastrointestinal system

A number of reports indicate that ORs play roles in a variety of cell types within the gastrointestinal system. To date, four reports have focused on enterochromaffin (EC) cells. One showed that OR2J3 is expressed in a pancreatic EC cell line, and that helional (an OR2J3 ligand) acts to alter calcium signaling and serotonin release (Kalbe et al., 2016b). Another paper identified four different ORs in microdissected human EC cells as well as in an EC cell line. The authors of that study found that ligands for these ORs altered both calcium signaling and serotonin release (Braun et al., 2007), whereas a separate group showed that Olfr78 is expressed in EC cells of the colon (Fleischer et al., 2015). Finally, a study from Kidd et al. (2008) investigated OR1G1 (as well as additional receptors, including T2R1) in FACS-sorted and neoplastic EC cells. Elsewhere in the gastrointestinal tract, researchers have investigated the role of OR51B4 in colorectal cancer. Weber et al. (2017) found that a novel ligand for OR51B4 had anti-proliferative, anti-migratory, and pro-apoptotic effects. In addition, OR1A1 has been shown to suppress PPAR-gamma expression in cultured hepatocytes (Wu et al., 2015), and Olr59 (a rat OR) was shown to be upregulated in the liver of F344 rats after triiodothyronine treatment (Matsubara et al., 2017). Although Olr59 is a rat OR, it has clear human (OR51E2) and murine (Olfr78) orthologs. Finally, the mouse OR Olfr544, which does not have a known human ortholog, has been examined for a potential role in the gastrointestinal tract. In 2015, one group showed that Olfr544 is expressed in alpha cells of mouse pancreatic islets, where it regulates glucagon secretion via calcium mobilization in response to its ligand, azelaic acid (Kang et al., 2015). In 2017, a second group showed that Olfr544 is expressed in liver and adipose tissue, where the authors proposed it contributes to a shift in fuel preference toward fats (Wu et al., 2017).

#### Genitourinary system

The first functional example of an OR expressed outside of the nose was a report in 2003 showing that OR17-4 was expressed in human sperm. The authors suggested that the ligand for this OR (bourgeonal) may help direct the sperm in the direction of the egg to aid in fertilization (Spehr et al., 2003). This landmark study was followed by a series of studies from the same group examining not only the role of OR17-4 (Spehr et al., 2004; Neuhaus et al., 2006), but additional ORs as well (Veitinger et al., 2011; Flegel et al., 2015). In 2016, a study using RNASeq found 91 OR transcripts in human sperm, although intriguingly, 22% of them were in an antisense formation, indicating that they may not code for a functional protein (Flegel et al., 2015). The role of ORs in the prostate (and, most often, prostate cancer) has also been investigated in several studies, many of which focused on “prostate-specific G-protein-coupled receptor” (PSGR), another name for OR51E2, which has been referenced several times above. PSGR/OR51E2 has been shown to play a role in inhibiting proliferation of prostate cancer cells (Neuhaus et al., 2009), a function that is thought to involve activation of TRPV6 (Spehr et al., 2011) and Pyk2 (Wiese et al., 2015). Similarly, PSGR/OR51E2 has been tied to cancer cell invasiveness (Sanz et al., 2014) and is a tumor antigen recognized by CD8+ T cells (Matsueda et al., 2012). PSGR/OR51E2 expression levels have been found to correlate with prognosis (Cao et al., 2015), and a mouse model of PSGR/OR51E2 overexpression suggests that this receptor does indeed play a role in tumor development (Rodriguez et al., 2014). There is also one report that the closely related receptor OR51E1 also inhibits growth of prostate cancer cells (Maßberg et al., 2016) and another that OR10H1 plays a potential role in bladder cancer (Weber et al., 2018). Finally, it has also been shown that ORs are expressed in the kidney and play functional roles in renal function. A study in 2009 reported that several ORs, along with G_olf_ and AC3, are expressed in the murine kidney, and that G_olf_ and AC3 localize to the macula densa (a chemosensory cell type) (Pluznick et al., 2009). Subsequently, it was reported that Olfr78 (the murine ortholog of OR51E2) localizes to juxtaglomerular cells in the kidney, where it acts to modulate renin secretion (Pluznick et al., 2013). Olfr1393 (a murine OR without a clear human ortholog) was reported to be expressed in renal proximal tubule cells, where it influences glucose handling. (Shepard et al., 2016). A number of other ORs (and taste receptors) have been identified in murine kidney (Rajkumar et al., 2014). ORs, along with G_olf_ and AC3, have also been found in the human proximal tubule cell line HK-2 (Kalbe et al., 2016b).

#### Cardiovascular system

ORs have been found in blood cells, where they are associated with tauopathy (Zhao et al., 2013) as well as chronic myelogenous leukemia (Manteniotis et al., 2016a,b). In addition, OR10J5 has been reported to play a role in angiogenesis (it is expressed in human aorta and coronary artery, as well as an endothelial cell line) (Kim et al., 2015). Another group reported that myocardial function is modulated via activation of an odorant receptor, OR51E1 (Jovancevic et al., 2017a).

#### Skin

In 2014, it was reported that exposure to odorants (such as Sandalore) induced ATP release from keratinocytes and thereby signaled to trigeminal neurons, implying that keratinocytes must have a way to sense the odorant (Sondersorg et al., 2014). Later that same year, it was shown that OR2AT4 expressed in human keratinocytes responds to Sandalore (Busse et al., 2014) and that activation of this receptor promotes cell proliferation and migration. These findings led to the hypothesis that OR2AT4 may play a role in wound healing. Subsequently, additional ORs (OR51B5 and OR2A4/7) have been identified in keratinocytes (Tsai et al., 2017), and OR51E2 has been identified in melanocytes (Gelis et al., 2016) and melanoma (Gelis et al., 2017).

#### Muscle

To date, the only OR characterized in muscle is MOR23 (Olfr16; a murine OR with a putative human ortholog of OR10J5). In 2009, it was shown that a soluble ligand for MOR23 is secreted by muscle cells and that loss of MOR23 leads to increased myofiber branching (Griffin et al., 2009). Subsequently, in 2016, the same group published work in which a transgenic mouse for MOR23 was crossed with dystrophic mice. They found that mechanical stress caused less damage to muscles from MOR23-overexpressing dystrophic mice than to those from control dystrophic mice (Pichavant et al., 2016).

#### Other

OR51E2 has been reported to play a role in regulating cell growth of retinal pigment epithelial cells (Jovancevic et al., 2017b). A landmark study in 2013 used next generation sequencing to profile the expression of ORs in 16 human tissues. The authors found that all tissues examined expressed at least one OR (Flegel et al., 2013). In a similar vein, researchers in 2015 generated a novel antibody for Olfr603 (murine OR with no clear human ortholog) and found that it is expressed in a number of different cell types, including vascular endothelium, smooth muscle, and migrating neural crest (Baker et al., 2015). Thus, it is clear that ORs are expressed in additional cell types and tissues where their functions have yet to be uncovered. Looking forward, it will be critical to continue to unmask the functions of these “ectopically” expressed ORs.

## Therapeutic potential

The majority of medications target GPCRs; however, over 30% of these receptors do not have a known endogenous ligand and therefore make ideal targets for future therapeutic interventions (Insel et al., 2012). Sensory receptors are located in many different tissue types and their expression and function are altered during disease states. Thus, they may represent new therapeutic targets for treating and altering disease progression.

### Bitter taste receptors (TAS2Rs)

Many disease processes throughout the body have the potential to be treated with TAS2R agonists.

#### Innate immunity

TAS2R function in the upper airway has been well characterized. TAS2Rs in mucociliary epithelial cells of the upper airway and nasal tissue appear to be directly involved in sinus disease. Patients with chronic rhinosinusitis who have gene allelic frequencies favoring dysfunctional TAS2R38 are at higher risk for sinus surgery than those who are homozygous for functional TAS2R38 alleles (Lee and Cohen, 2014b; Adappa et al., 2016). Studies have shown that ciliary beat frequency increases with TAS2R agonists (Shah et al., 2009) and TAS2R38s are present on neutrophils and lymphocytes that are active in binding bacterial proteins (Maurer et al., 2015).

#### Obesity

Bitterness inhibits hunger. Activation of enteroendocrine cells via TAS2Rs results in increased ghrelin levels, acutely increasing food intake; however, it also leads to decreased gastric emptying, which, in mice, decreases food intake in the long term (Janssen et al., 2011). In addition, bitter agonists administered to the stomach activate the nucleus tractus solitarii, and via vagal nerve activation, slow gastric emptying in human volunteers.(Glendinning et al., 2008; Avau et al., 2015) Mice that overexpress TAS2R16 (a receptor to which β-glucopyranosides selectively bind) and TAS2R38 receptors exhibited food avoidance behavior, providing a potential therapeutic target for appetite suppressant medications.

#### Smooth muscle relaxation

Activation of TAS2Rs leads to smooth muscle relaxation in many different tissue types, providing therapeutic targets to treat diseases like pulmonary hypertension, reactive airway disease (including asthma), and bladder spasms. Medications that target TAS2Rs have mitigated asthma symptoms (Sharma et al., 2017) and bladder spasms (Zhai et al., 2016) in animal models, but these studies have yet to be repeated in human trials. Studies showing vascular relaxation are limited to vessels in the brain and gastrointestinal tract; however, additional studies should be conducted to determine changes to peripheral and cardiac vasculature.

#### Neurodegenerative diseases

Little is known regarding the function of TAS2Rs in the CNS. TAS2R receptor mRNA expression is deregulated in Parkinson's disease, Alzheimer's disease, Creutzfeldt-Jakob disease, and schizophrenia (Ansoleaga et al., 2013, 2015; Garcia-Esparcia et al., 2013). Many different agonists are known to bind TAS2Rs (Meyerhof et al., 2010), but few are receptor-specific. Therefore their use presents high potential for side effects caused by unintended receptor activation. Future research should focus on receptor-specific therapeutic medications to limit such effects.

### Sweet and umami receptors (TAS1Rs)

These receptors also contribute to progression of pathologic conditions, such as airway inflammation and asthma, and metabolic diseases of the pancreas, such as diabetes. The most significant role that TAS1Rs might have in the future is treatment for gastrointestinal diseases and obesity.

#### Obesity

In contrast to bitter receptor activation, sweet sensation promotes food intake. Obesity decreases both bitter and sweet receptor expression in the duodenum and in areas of the murine brain involved in energy homeostasis. TAS1R3 is downregulated in the stomachs of obese patients (Widmayer et al., 2011). Obese mice exhibit decreased brain expression of TAS1R2, TRPM5, and the GPCR subunit Gα14. In addition, elevated glucose downregulates TAS1R2 and Gα14 and elevated leptin levels downregulate TAS1R3 are both consequences of obesity (Chao et al., 2016). These obesity effects suggest that TAS1R agonists may provide a therapeutic target to limit excess food intake.

#### Immunity

Like bitter taste receptors, TAS1Rs are also involved in bacterial recognition and immune cell function. Stimulation of the innate immune response by altering sweet receptor expression may benefit those chronically infected or unresponsive to traditional anti-bacterial medications. In addition, TAS1R3 agonists may be useful as anti-inflammatory medications because they inhibit the production of TNF-α and IL1β.

#### Neurodegenerative diseases

These receptors may play a role in cognitive diseases, including the initial stages of Alzheimer's disease, during which brain glucose metabolism decreases by ~45% (de la Monte et al., 2015). These taste receptors may hold potential as therapeutic targets to treat these diseases, but this possibility requires investigation.

### Photoreceptors

The discovery of nonvisual opsins in non-classic sensory organs prompts us to question whether these receptors can be engaged for therapeutic potential.

#### Obesity

The discovery that OPN4 receptors and TRPC channels in subcutaneous fat mediate a light-induced lipolytic activity suggests that blue-wavelength light might be used to reduce subcutaneous fat in a safe, noninvasive manner (Ondrusova et al., 2017). Since subcutaneous white adipose tissue is the main fat deposit in the human body, this could have big implications to aid in fat deposit regulation and the associated metabolic disorders.

#### Skin and hair health

Some applications of photoreceptors are already being explored for skin health, such as the use of directed light therapy on the skin to treat acne (Jung et al., 2015) or the recent suggestion of adding a blue light filter to broad spectrum sunscreen for the treatment of patients with hyperpigmentation (Boukari et al., 2015). Regarding these applications, OPN3 has been suggested to take part in melanogenesis, but no known photoreceptor has been associated yet with the acne reduction in response to blue or red light. With respect to hair health, recent studies showed OPN2 and OPN3 receptors are present in hair follicles. Furthermore, blue light exposure was shown to have a positive effect on hair growth, an effect mediated by OPN3. These results suggest that exposure to blue light may be a safe treatment for alopecia (Buscone et al., 2017).

#### Neurodegenerative diseases

The idea of using light to control the function of specific brain regions and to treat neurologic disorders has led to the field of optogenetics (Deisseroth, 2015). Optogenetic biology involves introducing viral-based expression vectors of wavelength-specific bacterial opsins into neurologic tissue to act as light-activated “molecular switches” that can alter membrane potential and induce physiologic responses. Fiber-optic cables targeted to specific regions of the brain or other tissue can serve as the switch activators. The limitations of this technology are (1) the need to transfect the light-activated switches using viral vectors and (2) the need for light-directing cables to be implanted in specific areas. With regard to the first limitation, the need for transfecting channels can be bypassed by recruiting the endogenously expressed OPNs in each tissue, or endogenous optogenetics. The second limitation of using fiber optic cables can also be circumvented. Whereas blue light, the wavelength that activates the OPN3 and 4 receptors, has very limited tissue penetrance, NIR light has the potential for deep tissue penetration, including bone. Upconverting nanocrystals, nanoparticles coated in rare earth lanthanides, can convert 2 photons of NIR light to 1 photon of blue light (Christ and Schaferling, 2015). The delivery of these crystals in tissues might therefore allow the use of NIR light to stimulate and activate endogenous OPN receptors by up-conversion to blue light (Chen et al., 2018). Given the expression of these OPN receptors in blood vessels and several brain tissues, this process may open up a potential light-based therapeutic path for the treatment of a wide variety of diseases.

#### Cardiovascular diseases

As discussed previously, the phototherapeutic approach of using directed blue light delivery along with GRK2 inhibition can effectively induce sustained vascular relaxation by activating OPN3 and OPN4, while preventing receptor desensitization by GRK2. This treatment has been demonstrated to alleviate both chronic and acute PH in rat models, though its application could be extended to other cardiovascular diseases characterized by abnormal vasoconstriction (Barreto Ortiz et al., 2018). Additionally, this phototherapy has the potential of inducing vasodilation in acute vascular obstruction events easily and safely, possibly buying precious time for a patient experiencing a cardiovascular episode such as a stroke or heart attack and allowing blood flow to the affected areas until surgical procedures to remove the obstruction can be performed. Further research on the clinical applicability of phototherapy in vascular relaxation is therefore crucial.

### Olfactory receptors

As ORs comprise the largest gene family in the genome, opportunities likely exist to leverage these receptors to modify physiologic and pathophysiologic processes. Such aspirations are currently limited by the fact that many ORs remain orphan receptors, with no known ligand. Because the most obvious strategy for commandeering these receptors for therapy would be to use agonists or antagonists, it is necessary that ligands be clearly identified for each of the ORs. Although past efforts to identify ligands have been hampered by technical hurdles regarding OR trafficking *in vitro*, recent studies have made progress in this area (Saito et al., 2004; Shepard et al., 2013; Peterlin et al., 2014), offering reason for optimism. ORs may lend themselves to being leveraged therapeutically in several potential areas, but we should caution that in all areas, future work is warranted to establish the feasibility and efficacy of potential interventions.

#### Asthma

OR51E2 has been shown to modulate proliferation and cytoskeletal remodeling in ASM from both asthmatics and non-asthmatics, implying that modulation of OR51E2 signaling may be beneficial in asthma (Aisenberg et al., 2016).

#### Cancer

A number of studies in several different systems have pointed to a potential role of ORs in controlling the growth of cancer cells. Hence, modulating OR signaling might offer the possibility to inhibit cancer cell growth. OR51E2/PSGR has been shown to inhibit the growth of prostate cancer cells (Neuhaus et al., 2009; Spehr et al., 2011; Wiese et al., 2015). However, the same OR has also been shown to stimulate cancer cell invasiveness (Sanz et al., 2014), and overexpression of PSGR in mouse tumors suggests a role for this receptor in tumor development (Rodriguez et al., 2014). Thus, the utility of modulating OR51E2/PSGR in cancer is currently unclear. OR51E1 also has been reported to inhibit prostate cancer cell growth (Maßberg et al., 2016). In addition, OR2J3 activation induced apoptosis and inhibited cell proliferation in a non-small-cell lung cancer cell line (Kalbe et al., 2017); OR51B4 activation was found to have anti-proliferative, anti-migratory, and pro-apoptotic effects in colorectal cancer (Weber et al., 2017); and OR10H1 has been reported to play a role in bladder cancer (Weber et al., 2018).

#### Metabolism

Olfr544 has been reported to play a role in the regulation of glucagon secretion (Kang et al., 2015) and in steering fuel preference toward fats (Wu et al., 2017). In addition, Olfr1393 modulates renal glucose handling by modulating Sglt1 (Shepard et al., 2016). Of note, Sglt1 is similar in function to Sglt2, a current drug target used to reduce blood glucose in type 2 diabetes.

#### Infertility

The suggestion that bourgeonal may act through an OR to help direct the sperm in the direction of the egg to aid in fertilization (Spehr et al., 2003) implies that modulation of this pathway may be efficacious in infertility.

#### Hypertension

Olfr78 increases renin secretion by the kidney and also plays a role in modulating vascular tone (Pluznick et al., 2013), implying that modulation of this receptor could be efficacious in hypertension.

#### Heart failure

Activation of OR51E1 has been associated with negative chronotropic and inotropic effects on the heart (Jovancevic et al., 2017a).

#### Wound healing

Activation of OR2AT4 has been shown to promote cell proliferation and migration in keratinocytes (Busse et al., 2014), implying that it may play a role in wound healing.

#### Muscular dystrophy

When transgenic mice for MOR23 were crossed with dystrophic mice, mechanical stress caused less damage to the muscles from dystrophic mice with elevated MOR23 than to muscles from dystrophic mice with normal MOR23 levels (Pichavant et al., 2016).

## Conclusion

In conclusion, receptors traditionally believed only to identify and interpret light, sound, and taste also contribute to normal functions within many if not all other organ systems. Determining the function of sensory receptors in these organ systems and their roles under normal and pathophysiologic conditions has become a primary research focus with the potential to identify novel therapeutic targets to treat and possibly cure many medical diseases.

## Author contributions

ND, SB, JP, and DB contributed the underlying data, designed and created the Tables and Figures, and edited and wrote the final manuscript. ND, SB, and JP primarily wrote the sections on Taste, Photo, and Olfactory receptors, respectively. DB edited the final manuscript, contributing sections for novel therapeutic potential.

### Conflict of interest statement

The authors declare that the research was conducted in the absence of any commercial or financial relationships that could be construed as a potential conflict of interest.
